# Prostate Cancer: Insights into Disease Progression and Therapeutic Challenges

**DOI:** 10.3390/ijms25052451

**Published:** 2024-02-20

**Authors:** Debanjan Chakroborty, Ajay Pratap Singh

**Affiliations:** 1 Department of Pathology, University of South Alabama, Mobile, AL 36617, USA; 2 Cancer Biology Program, Mitchell Cancer Institute, University of South Alabama, Mobile, AL 36604, USA; 3 Department of Biochemistry and Molecular Biology, University of South Alabama, Mobile, AL 36688, USA

Prostate cancer (PCa) is the second most common cancer and the fifth highest cause of cancer-related death among men in the world [1]. In the United States, it is the foremost cause of cancer-associated death among men [2]. Despite significant advancements in diagnosis and treatment, PCa remains a major health burden, and its incidence and fatality continue to rise owing to its heterogeneous nature, leading to variable prognoses, a lack of specific early diagnosis biomarkers, and the failure of most therapies in the long term [2,3]. In addition, disproportionate disease burden and treatment outcomes are reported in patients of different racial backgrounds, adding additional layers of complexity to the already complex PCa landscape [4,5]. Undoubtedly, a clear understanding of PCa pathology and pathobiology is critical for developing novel and improved approaches to diagnosis, prognosis, and treatment. This Special Issue was developed with an overarching goal of bringing a deeper understanding to readers about the current state of PCa-related research to help instigate new directions for future explorations through collaborative research. With four original research and two review articles, this Special Issue comprehensively addresses the diverse spectrum of disease biology together with molecular and metabolic alterations and their impact on disease outcomes in different patient populations.

Intra- and inter-tumor heterogeneity makes PCa management a challenging task. While localized PCa tends to have an excellent prognosis, patients with metastatic PCa often exhibit therapeutic resistance to primary and subsequent generation therapies stemming from genetic heterogeneity and metabolic adaptations [6]. The lack of specific biomarkers poses a significant challenge in accurately assessing the future course of tumor progression and response to therapies. Studies to date have identified several biomarkers, such as Ki 67, androgen receptor variant V7 (ARv7), C-MYC, and the loss of PTEN, for disease prognosis. However, despite having potential, these biomarkers do not have the necessary approval for clinical application because of their lack of ability to predict disease progression in a predictive manner [7,8]. Therefore, additional efforts are warranted to identify new biomarkers that can more precisely predict the progression and responsiveness of PCa to available therapies. In their original work, Ryabov et al. determined how the isolation and early culture of PCa cells from patients paired with appropriate normal controls could serve as a valuable tool for identifying disease-specific biomarkers for localized PCa. Their work also identifies how these isolated cell cultures from patients could be used as alternative preclinical models in future studies.

Prostate tumors typically exhibit a range of metabolic changes compared to normal prostatic tissues, which influences their progression and treatment outcomes [9]. Therefore, understanding metabolic changes is crucial to improving our knowledge of PCa development and is beneficial for developing new therapeutic approaches. In the past, glycolysis was considered a primary energy-producing metabolic process in cancer cells. However, emerging research suggests that mitochondrial metabolism also significantly determines malignant transformation and therapeutic outcomes in PCa [10]. The metabolism of glandular prostatic epithelial cells in the prostate peripheral zone is unique. Malignant transformation shows significant metabolic deviation resulting in increased citrate oxidation in these cells, which is attributable to the reduction in zinc accumulation in these cells [11]. Zhang et al. demonstrated in their original research work how inefficient mitochondrial energy transfer interferes with citrate oxidation and promotes the neoplastic growth of PCa cells. Using freshly collected, paired malignant and nonmalignant prostate tissues, they identified a dominant mitochondrial succinate CII oxidation process in PCa and reported that CI oxidation is impaired in advanced PCa. These findings represent a significant shift in our understanding of PCa metabolism by indicating that PCa cells are more reliant on CII-driven oxidative flux than CI activity. The clinical application of this observation is profound since manipulations of mitochondrial alteration offer promising avenues for new intervention and improved therapeutic outcomes.

Androgen and androgen receptors (ARs) play an essential role in the development and progression of PCa. Extensive research has highlighted the importance of the AR axis in disease progression and the effect of manipulating it on therapeutic response [12,13]. In their original research article, Chang et al. explored the intricate crosstalk between AR and NF-ĸB activation and its impact on the growth and metastasis of PCa. The involvement of mucosa-associated lymphoid tissue 1 (MALT1) and its regulation by the full-length androgen receptor (ARFL) and its splice variant, ARv7, in androgen-sensitive and castration-resistant PCa cells were determined in this study. Their findings not only reveal a novel molecular pathway involving androgen/ARFL/MALT1/NF-κB signaling but also provide a new dimension to our understanding of the diverse roles of ARs and their variants in PCa progression. Although inhibition of the AR axis is considered the cornerstone of PCa treatment, patients develop insensitivity to androgen manipulation over time due to sustained AR signaling through alternate ligand-independent pathways or the development of bypass mechanisms [14]. The review article by Tortorella et al. offers insights into the involvement of bypass pathways and how, together with ARs, they play crucial roles in PCa progression. Here, they discuss the important roles played by the PI3/AKT/mTOR pathway in manipulating the AR axis and the development of therapeutic resistance.

Androgen deprivation therapy (ADT), while helping to curb PCa growth, also has some deleterious effects on patients’ overall health. Its prolonged use can cause problems such as bone loss, osteoporosis, and cardiovascular complications like arrhythmia and hypertension [15,16]. It is, therefore, essential to address these related problems for improved disease management. Suominen et al. explored the possibility of combining radium 223 with AR inhibition and studied the outcome in preclinical PCa models. Their study findings indicate that the combined regimen has better results than single-agent therapy and does not compromise bone health/integrity. Bone strength is often compromised in PCa patients because of advanced age, hypogonadism, and PCa cell metastasis [15]. This study, therefore, has significant clinical implications as preserving bone integrity may improve the quality of life of the patients.

Cardiovascular diseases (CVDs) or cardiovascular complications (CVCs) often interfere with PCa outcomes and pose significant challenges to patient management [16,17,18]. Studies have highlighted a strong connection between these two diseases where patients with pre-existing CVCs/CVDs are reported to have increased PCa risk or are presented with advanced and fatal forms of the disease [15,16,17,18]. Additionally, a number of cardiovascular issues were reported in PCa patients receiving first- or second-generation ADT [17,18,19,20]. However, despite these reports, the connection between these two diseases is still elusive and needs further investigation. Kakkat et al. discussed the association between PCa and CVDs by analyzing genomic data extracted from publicly available datasets of mCRPC patients from several clinical trials. This information is highly valuable because almost 50% of PCa patients die of CVDs [17,18]. The article provides useful information by pinpointing shared metabolic pathways between PCa and CVDs and identifying new molecular connections between these two diseases.

In summary, we hope that this Special Issue will serve as an invaluable resource for basic, translational, and clinical researchers by offering collective insight into the disease pathology, pathobiology, and mechanisms of therapeutic resistance. The collection highlights various aspects of PCa, from unraveling the metabolic intricacies that influence disease progression to deciphering the molecular mechanisms that aid in the development of therapeutic resistance. We believe that this comprehensive compilation of information will not only help expand the horizon of our knowledge but will also be instrumental in fueling further research to better understand the complex PCa landscape.
